# Gut microbiota-derived tryptophan metabolite indole-3-carboxaldehyde enhances intestinal barrier function via aryl hydrocarbon receptor/AMP-activated protein kinase signaling activation

**DOI:** 10.5713/ab.25.0225

**Published:** 2025-07-11

**Authors:** Donglin Shi, Yong Cui, Haiping Liang, Qing Wei, Jianzhen Huang, Ji Cao

**Affiliations:** 1 College of Animal Science and Technology, Jiangxi Agricultural University, Nanchang, China; 2 State Key Laboratory of Natural Medicines, China Pharmaceutical University, Nanjing, China

**Keywords:** Aryl Hydrocarbon Receptor/AMP-activated Protein Kinase Signaling, Autophagy, Colitis, Indole-3-carboxaldehyde, Intestinal Barrier, Mitochondrion

## Abstract

**Objective:**

Intestinal inflammatory diseases significantly affect animal health, primarily by disrupting intestinal barrier function. Indole-3-carboxaldehyde (IAld), a key metabolite of tryptophan derived from gut microbiota, exhibits protective properties against intestinal inflammatory diseases. The regulatory mechanism by which IAld modulates intestinal barrier function requires further investigation.

**Methods:**

An intestinal epithelial cell injury model was established by tumor necrosis factor-alpha (TNF-α) stimulation, alongside a mouse colitis model induced by dextran sulfate sodium (DSS) administration. Intestinal barrier function was assessed by immunoblotting, immunofluorescence, *in vitro* permeability assays, and histopathological analysis. Mitochondrial integrity and function were evaluated using JC-1 staining and transmission electron microscopy. Additionally, key components of the aryl hydrocarbon receptor (AhR)/AMP-activated protein kinase (AMPK) signaling pathway were analyzed using immunoblotting, immunofluorescence, and immunoprecipitation techniques.

**Results:**

Our findings demonstrate that IAld treatment significantly enhanced tight junction protein expression in intestinal epithelial cells and effectively attenuated TNF-α-induced intestinal barrier injury. IAld activated cellular AMPK signaling, promoting autophagy, maintaining mitochondrial homeostasis, and ultimately improving intestinal barrier function. Importantly, the activation of AMPK signaling by IAld was found to be dependent on the AhR, as evidenced by the AhR-specific inhibitor CH-223191, which abolished both IAld-induced AMPK activation and enhancement of intestinal barrier integrity. Furthermore, *in vivo* experiments confirmed that IAld ameliorated intestinal barrier dysfunction and mitochondrial damage in DSS-induced colitis mice, whereas pharmacological inhibition of AMPK largely abrogated these protective effects.

**Conclusion:**

Our findings demonstrate that IAld effectively preserves intestinal barrier integrity, highlighting its potential application in the treatment of intestinal inflammatory diseases in both animals and humans.

## INTRODUCTION

The intestine serves as the primary site for food digestion and nutrient absorption. To optimize nutrient uptake, the intestinal epithelium must maintain selective permeability while preserving tight junction (TJ) integrity—a critical barrier function that prevents pathogenic microorganisms from infiltrating the body [1]. Intestinal permeability is primarily regulated by TJ proteins such as zona occludens-1 (ZO-1) and occludin (Ocln), which seal intercellular gaps to form a contiguous monolayer of intestinal epithelial cells [1]. Excessive pro-inflammatory cytokines (*e.g.* tumor necrosis factor-alpha [TNF-α]) can reduce TJ protein expression and trigger mucosal hyperpermeability [2], a dysfunction implicated in inflammatory disorders such as post-weaning diarrhea in piglets [3] or inflammatory bowel disease in humans [4]. Thus, targeted restoration of intestinal barrier function represents a promising therapeutic strategy for gut-related inflammatory conditions.

Gut microbiota-derived metabolites serve as key signaling molecules that mediate microbiota-host interactions [5]. Recently, therapies based on gut microbiota-derived metabolites have emerged as a promising strategy for treating intestinal inflammatory diseases [6]. Indole-3-carboxaldehyde is (IAld) primarily synthesized by gut microbiota (*e.g. Lactobacillus reuteri*) through tryptophan (Trp) metabolism [7]. As an endogenous ligand of aryl hydrocarbon receptor (AhR), IAld can alleviate colitis in mice via AhR-dependent mechanisms, primarily by maintaining intestinal homeostasis [7]. Furthermore, IAld enhances intestinal barrier function in weaned piglets by promoting intestinal stem cell expansion [8]. Our recent study demonstrated that IAld can attenuate lipopolysaccharide (LPS)-induced intestinal inflammatory injury through AhR activation [9], highlighting its therapeutic potential for intestinal inflammatory disorders.

AMP-activated protein kinase (AMPK), a central energy sensor and regulator in mammalian systems, enhances TJ protein expression by suppressing reactive oxygen species generation, thereby protecting against LPS-induced barrier dysfunction [10]. Furthermore, AMPK modulates the distribution of TJs within the intestinal epithelium [11]. AhR similarly plays a pivotal role in barrier maintenance, with its intestinal protective effects well-documented [6,12]. However, the mechanistic crosstalk between AhR and AMPK signaling pathways remains unclear, and whether IAld improves intestinal barrier function through AhR/AMPK-mediated mechanisms requires further investigation.

In this study, we elucidated the molecular mechanism by which IAld regulates intestinal barrier function. Our results demonstrate that IAld potently activates the AhR/AMPK signaling pathway, which in turn enhances autophagy and restores mitochondrial homeostasis in intestinal epithelial cells. These coordinated cellular responses collectively improve intestinal barrier function, thereby mediating IAld’s anti-colitis effects in murine models. Based on these mechanistic insights, we conclude that IAld ameliorates intestinal barrier dysfunction via AhR/AMPK signaling activation, highlighting its potential as a novel therapeutic candidate for intestinal inflammatory diseases in animals.

## MATERIALS AND METHODS

### Establishment of mouse colitis model and indicated treatments

Fifteen 6–8 weeks old male C57BL/6 mice (18–20 g; Beijing Vital River Laboratory Animal Technology) were acclimatized for 7 days under standard conditions (24±°C, 12 h light/dark cycle) with *ad libitum* access to food and water. To induce colitis, mice were given 2.5% (w/v) dextran sodium sulfate (DSS) into drinking water for 7 days followed by regular water for the next 3 days.

The specific animal experiment process was as follows: Mice with DSS administration were randomly divided into 3 groups: DSS group (n = 5), DSS+IAld (50 mg/kg body weight) treatment group (n = 5), Compound C (1 mg/kg body weight)+DSS+IAld (50 mg/kg body weight) treatment group (n = 5). The Compound C (suspended in 0.5% carboxymethylcellulose [CMC]) gavage treatments started 3 days prior to DSS exposure once per day, IAld (suspended in 0.5% CMC) gavage treatment started from DSS administration until day 7, DSS group mice received with the equal vehicle (0.5% CMC). Body weight change was recorded daily. At the end of the experiment point (day 10), mice were humanely sacrificed by cervical dislocation, the colons samples were collected for subsequent analysis. The evaluation of the disease activity index (DAI) was performed as previously described [13]. The specimens of the colon were fixed with 10% formalin for H&E and alcian blue staining.

### Reagents and antibodies

IAld and dimethyl sulfoxide (DMSO) were provided by Sigma-Aldrich. The fetal bovine serum (FBS) and Dulbecco’s modified Eagle’s medium (DMEM) were obtained from Gibco. The MCE provided inhibitors including hydroxychloroquine (HCQ), Compound C, and CH-223191. Recombinant human TNF-α protein was purchased from Bioword. DSS with molecular weights of 36,000 to 50,000 was purchased from MP Biomedicals. The rabbit anti-ZO-1 (BS71522), Ocln (BS72035), p-AMPK (BS5003), AMPK (BS1009), LC3 (BS66159), TFEB (BS80335), GAPDH (AP0063), β-Tubulin (AP0064), and goat anti-rabbit IgG (H+L)-HRP (BS13278) antibodies were obtained from Bioword. The rabbit anti-AhR (A4000) antibody was obtained from ABclonal. The goat anti-rabbit IgG (H+L) FITC (GB22303) and goat anti-rabbit IgG (H+L) Cy3 (GB21303) antibodies were obtained from Servicebio.

### Cell culture

The intestinal epithelial cells Caco2 were generously provided by Prof. Haitian Ma (Nanjing Agricultural University). Cells were maintained in DMEM culture medium containing 10% fetal calf serum in a 37°C humidified incubator with 5% CO_2_ and passaged every 2–3 days.

The extraction and cultivation of mouse colonoids were performed as previously described with minor modifications [14]. Briefly, take colon tissue from 6-week-old C57BL/6 mice, clean the intestinal segment with D-hanks buffer, remove fat, mucosa, etc., cut the tissue into small pieces, and then add collagenase I solution for digestion until most of the crypts fall off. After cleaning the intestinal crypts, use matrigel (BD Bioscience/Corning) to resuspend cells, and place them in a 24-well cell culture plate at a rate of 15 μL/well. After the matrix glue solidifies, OGM medium (STEMCELL) was overlaid and refreshed every 2 days.

### Construction of intestinal epithelial cell injury model

The Caco2 cells were stimulated with TNF-α (10 ng/mL) for 24 h to establish an intestinal epithelial cell injury model, as previously described [15]. The destruction of TJs and the increase in intestinal epithelial permeability indicated the successful construction of the *in vitro* model.

### Transmission electron microscopy

The colon tissues were fixed with 2.5% glutaraldehyde (Servicebio), followed by PBS rinsing, acid fixation, gradient dehydration, embedding, ultra-thin sectioning, and 3% uranyl acetate lead citrate staining. The images of intestinal epithelial morphology and mitochondria microstructure were acquired using the Hitachi HT7700 transmission electron microscopy (TEM).

### Western blotting

Total proteins of cells or colon tissue were extracted with RIPA lysis buffer (Servicebio) as per the manufacturer’s protocol. The concentrations of proteins were determined by the Pierce BCA protein assay kit (Beyotime). Western blotting (WB) was performed as previously described [9]. Briefly, protein samples were separated by 10% SDS-PAGE and then transferred onto the 0.45 μm PVDF membranes (GE Healthcare). The membranes were blocked with 5% non-fat milk for 1 h at room temperature. After washing with TBST, the membranes were incubated with the indicated primary antibody (dilution ratio of 1:1,000) or internal reference antibody β-Tubulin or GAPDH (dilution ratio of 1:10,000) at 4°C overnight. Subsequently, the membranes were incubated with HRP goat anti-rabbit IgG (dilution ratio of 1:10,000) at 37°C for 1 h. The protein bands were visualized using a clarity ECL chemiluminescent substrate (Vazyme Biotech) and quantified with ImageJ software (ver. 1.48).

### Immunoprecipitation

The immunoprecipitation (IP) analysis was conducted as previously reported [13]. Briefly, cells were harvested and lysed with NP-40 buffer containing protease inhibitor cocktail. The cell lysates were immunoprecipitated with anti-p-AMPK antibody, and Bioepitope^R^ protein A+G agarose beads (Bioword). The IP results were analyzed by WB.

### Molecular docking

Molecular docking was performed as previously described [16]. Briefly, the three-dimensional (3D) structure of the AhR protein was obtained from the RCSB Protein Data Bank (RCSB PDB) (https://www.rcsb.org/), and the 3D structure of IAld (CID 10256) was obtained from PubChem (https://pubchem.ncbi.nlm.nih.gov/). The protein structure was processed by dehydration, hydrogenation, and other treatments using PyMOL software (ver. 2.6.0). Subsequently, AutoDock software (ver. 4.2.6) was used to perform molecular docking between the AhR protein and IAld. Finally, the molecular docking results were visualized using PyMOL software.

### Immunofluorescence

The immunofluorescence (IF) analysis was performed as previously described [9]. Briefly, the fixed tissue or cells were incubated with the specific primary antibody, followed by incubation with the fluorescently labeled secondary antibodies. The cells were dyed with DAPI and analyzed using a Zeiss LSM 710 confocal microscope (Jena).

### Mitochondrial membrane potential detection

The mitochondrial membrane potential detection was performed as previously described [9]. In brief, the mitochondrial membrane potential was detected using the JC-1 fluorescent probe (Servicebio) following the instructions, respectively. In brief, the Caco2 cells were loaded with the JC-1 fluorescent probe for 20–30 min at 3°C. After incubation and washing, the fluorescence intensity was immediately detected using a Zeiss LSM 710 confocal microscope (Jena).

### *In vitro* intestinal permeability analysis

*In vitro* intestinal permeability analysis was performed as previously described [9]. Briefly, the Caco2 cells were seeded onto the upper chambers of 24-well Transwell plates (0.4 μm pore polyester membranes; Jet Biofil) until a stable monolayer was formed. After the specified treatments, FITC-Dextran (FD-4; MW 4000; Sigma-Aldrich) solution (1 mg/mL, 0.2 mL) was added to the upper chambers. After 24 h of incubation, samples were collected from the bottom chamber and use the fluorescence 96 wells plate reader (excitation wavelength 480 nm, emission wavelength 525 nm) to detect the FD4 flux.

### Statistical analysis

All data were expressed as mean±standard error of the mean from at least three independent experiments. One-way analysis of variance (ANOVA) and unpaired Student’s t-test (two-tailed) were performed by GraphPad Prism software (ver. 8.0.2) to compare the significant differences among different treatment groups. One-way ANOVA was used to calculate the differences between multiple groups (more than three groups), and Student’s t-test was used to calculate the differences between two groups. Note: p<0.05 indicates a significant difference, while p<0.01 indicates an extremely significant difference.

## RESULTS

### Indole-3-carboxaldehyde prevents tumor necrosis factor-alpha-induced damage to the intestinal epithelial barrier

**Figure 1:**
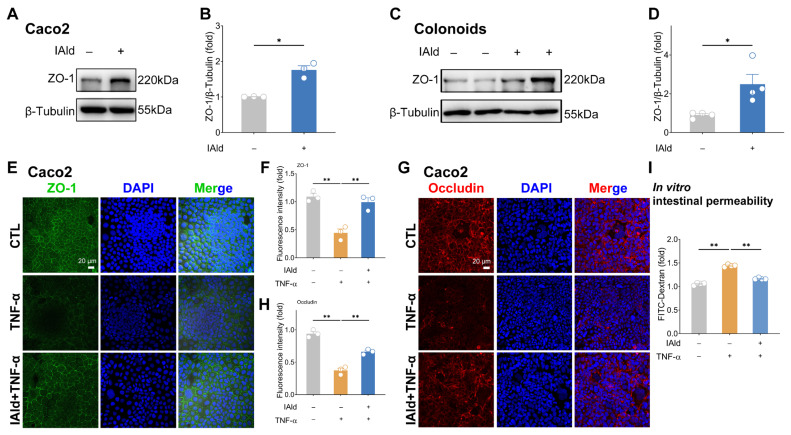
IAld prevents TNF-α-induced damage to the intestinal epithelial barrier. (A, B) Caco2 cells were treated with IAld (0, 10 μM) for 24 h, the ZO-1 protein expression level was measured by western blotting and quantified by Image J software. (C, D) Mouse colonoids were treated with IAld (0, 10 μM) for 24 h, and the ZO-1 protein expression levels were measured by western blotting and quantified by Image J software. (E, F) Caco2 cells were treated with IAld and stimulated with TNF-α (10 ng/mL) for 24 h. The ZO-1 protein expression and cell membrane distribution were analyzed by immunofluorescence and quantified by Image J software, scale bar = 20 μm. (G, H) The occludin protein expression was analyzed by immunofluorescence and quantified by Image J software, scale bar = 20 μm. (I) Caco2 cells were treated with IAld and stimulated with TNF-α (10 ng/mL) for 24 h, and the FITC-Dextran (FD-4) leakage level was detected to reflect the permeability of intestinal epithelial cells. Data are presented as means±SEM (n = 3 or 4). * p<0.05, ** p<0.01, compared with the respective control. ZO-1, zona occludens-1; TNF-α, tumor necrosis factor-alpha; IAld, indole-3-carboxaldehyde; SEM, standard error of the mean.

To investigate the effect of IAld on intestinal barrier function, we first treated Caco2 cells and mouse colonoids with a physiological concentration of IAld (10 μM; no cytotoxic was observed). As shown in Figures 1A–1D, IAld significantly upregulated the TJ ZO-1 protein expression in both Caco2 cells and mouse colonoids, demonstrating its regulatory role in intestinal barrier function. Given that TNF-α is a key pro-inflammatory cytokine implicated in intestinal inflammatory diseases and known to suppress TJ protein expression and induce intestinal barrier injuries [17], we employed a TNF-α induced intestinal epithelial cell injury model to further assess IAld’s protective effects. As shown in Figures 1E–1H, TNF-α (10 ng/mL, the concentration was selected based on published literature [18]) markedly impaired the distribution of TJ proteins (ZO-1 and Ocln), whereas IAld treatment effectively restored their normal localization. Furthermore, our *in vitro* Transwell permeability assays revealed that TNF-α stimulation significantly elevated FD-4 leakage (reflecting increased permeability), which was substantially attenuated by IAld (Figure 1I). These above results suggest that IAld can promote TJ integrity in intestinal epithelial cells under both physiological and inflammatory conditions.

### Indole-3-carboxaldehyde exerts intestinal barrier protection by activating AMP-activated protein kinase signaling

**Figure 2:**
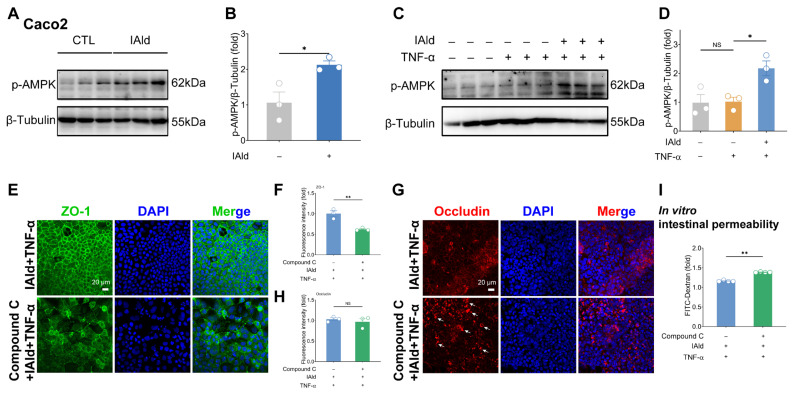
IAld exerts intestinal barrier protection by activating AMPK signaling. (A, B) Caco2 cells were treated with IAld (0, 10 μM) for 24 h, the p-AMPK protein expression levels were measured by western blotting and quantified by Image J software. (C, D) Caco2 cells were treated with IAld and stimulated with TNF-α (10 ng/mL) for 24 h, the p-AMPK protein expression levels were measured by western blotting and quantified by Image J software. (E, F) Caco2 cells were treated with IAld+TNF-α in the presence or absence of the AMPK-selective inhibitor Compound C (10 μM) for 24 h, and the ZO-1 protein expression and cell membrane distribution were analyzed by immunofluorescence and quantified by Image J software, scale bar = 20 μm. (G, H) The occludin protein expression and cell membrane distribution were analyzed by immunofluorescence and quantified by Image J software, scale bar = 20 μm. The white arrow indicates abnormal distribution of occludin on the cell membrane. (I) After the indicated treatment, the leakage of FD-4 induced by TNF-α-stimulation was measured. Data are presented as means±SEM (n = 3 or 4). * p<0.05, ** p<0.01, compared with the respective control; NS: no significance between the indicated groups. AMPK, AMP-activated protein kinase; TNF-α, tumor necrosis factor-alpha; IAld, indole-3-carboxaldehyde; ZO-1, zona occludens-1; SEM, standard error of the mean.

AMPK has been reported to play a critical role in regulating intestinal barrier function [11]. To explore whether IAld modulates this pathway, we examined its effect on AMPK signaling in Caco2 cells. Our results showed that IAld treatment significantly enhanced phosphorylation of AMPK in both normal Caco2 cells and TNF-α treated Caco2 cells (Figures 2A–2D), while having no significant effect on total AMPK protein expression (Supplements 1A–1D). These findings clearly demonstrate that IAld can activate the AMPK signaling pathway in intestinal epithelial cells.

To investigate the functional significance of AMPK activation in IAld-mediated protection, we employed the AMPK-selective inhibitor Compound C. Pretreatment with Compound C abolished IAld’s ability to maintain the expression and distribution of TJ protein ZO-1 (Figures 2E, 2F). Although Compound C did not affect Ocln protein levels, it disrupted IAld-induced maintenance of Ocln distribution, resulting in characteristic punctate aggregation (Figures 2G, 2H). Consistent with these observations, Compound C also reversed IAld’s protective effect on intestinal barrier function in our permeability assays (Figure 2I). Notably, AMPK inhibition triggered cell apoptosis (evidenced by nuclear condensation and fragmentation in DAPI staining; Figures 2E, 2G), which may further exacerbate intestinal barrier dysfunction [19]. Collectively, these above results suggest that IAld exerts intestinal barrier protection by activating AMPK signaling.

### Indole-3-carboxaldehyde induces autophagy and maintains mitochondrial function in Caco2 cells

**Figure 3:**
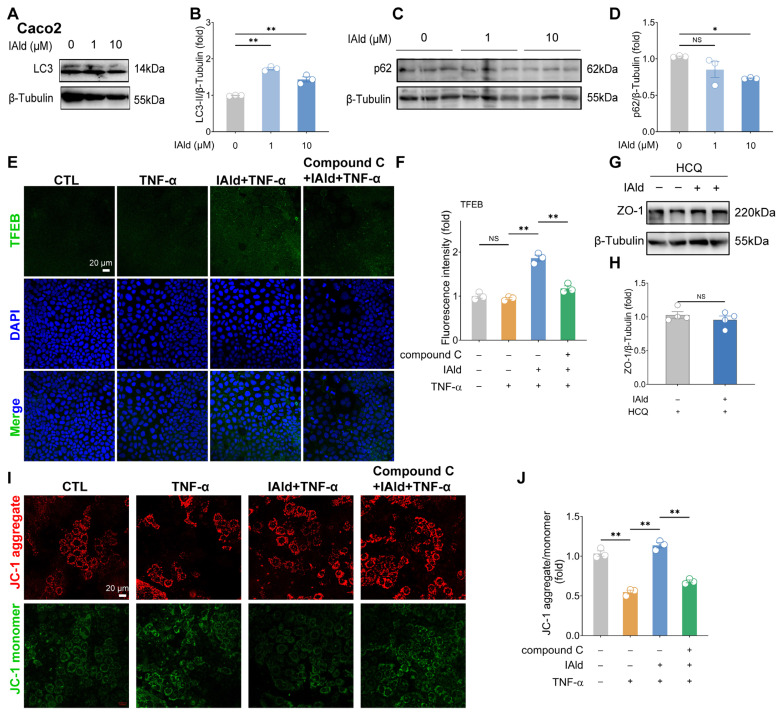
IAld induces autophagy and maintains mitochondrial function in Caco2 cells. (A, B) Caco2 cells were treated with IAld (0, 1, 10 μM) for 24 h, the LC3 protein expression levels were measured by western blotting and quantified by Image J software. (C, D) Caco2 cells were treated with IAld (0, 1, 10 μM) for 24 h, the p62 protein expression levels were measured by western blotting and quantified by Image J software. (E, F) Caco2 cells were treated with IAld+TNF-α in the presence or absence of the AMPK-selective inhibitor Compound C (10 μM) for 24 h, and the TFEB protein expression level was analyzed by immunofluorescence and quantified by Image J software, scale bar = 20 μm. (G, H) Caco2 cells pre-treated with HCQ (10 μM) were exposed to IAld (10 μM) for 24 h, and the ZO-1 protein levels were analyzed by western blotting and quantified by Image J software. (I, J) After indicated treatments, the cell mitochondrial membrane potential level was indicated by JC-1 staining and analyzed by immunofluorescence. The fluorescence intensity of the JC-1 monomer and aggregate was quantified by Image J software, scale bar = 20 μm. Data are presented as means±SEM (n = 3). ** p<0.01, compared with the respective control; NS: no significance between the indicated groups. IAld, indole-3-carboxaldehyde; TNF-α, tumor necrosis factor-alpha; ZO-1, zona occludens-1; AMPK, AMP-activated protein kinase; HCQ, hydroxychloroquine; SEM, standard error of the mean.

Autophagy is a key intracellular signal for maintaining cellular homeostasis, with AMPK serving as the upstream signal of autophagy induction [20]. Given the activation effect of IAld on AMPK signaling, we hypothesized that IAld could also enhance the autophagy level in intestinal epithelial cells. Using LC3, a typical biomarker of autophagy whose LC3-II form reflects autophagic activity [21], we observed that IAld (1 or 10 μM) significantly increased the LC3-II protein expression level (Figures 3A, 3B). Moreover, 10 μM IAld can markedly reduce the expression level of autophagy receptor protein p62 (another key biomarker of autophagy) in Caco2 cells (Figures 3C, 3D). Since the AMPK-TFEB pathway is a classic autophagy induction mechanism [22], we examined TFEB activation and found IAld promoted this process, an effect that was blocked by Compound C treatment (Figures 3E, 3F). The results confirm that IAld can induce the occurrence of autophagy in intestinal epithelial cells.

To determine the role of autophagy in IAld-induced TJ barrier enhancement, we employed autophagy inhibitor HCQ, which prevented IAld-mediated improvement of ZO-1 protein expression (Figures 3G, 3H). Furthermore, JC-1 staining revealed that IAld protected against TNF-α-induced mitochondrial damage, while Compound C abolished this protective effect (Figures 3I, 3J). Collectively, these findings demonstrate that IAld can promote intestinal barrier function through AMPK-dependent autophagy induction and mitochondrial maintenance.

### Indole-3-carboxaldehyde activates AMP-activated protein kinase signaling pathway through aryl hydrocarbon receptor

**Figure 4:**
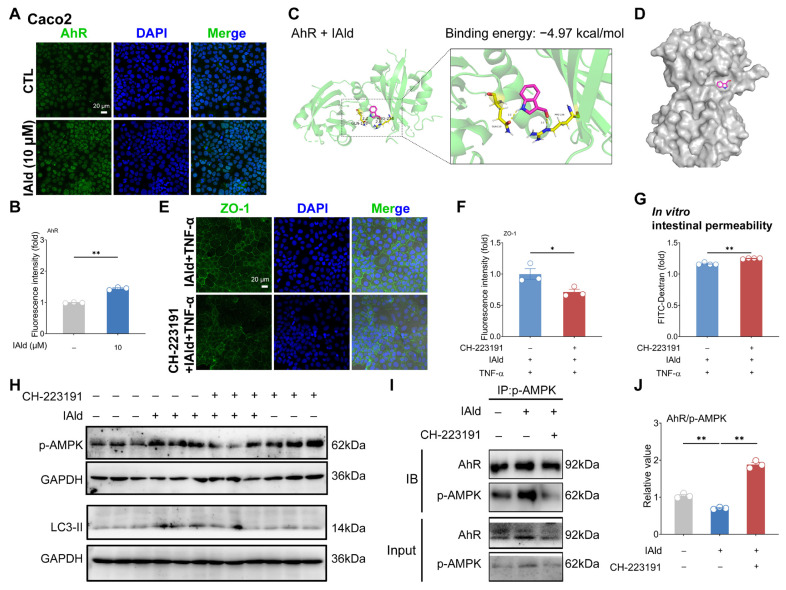
IAld activates AMPK signaling pathway through AhR. (A, B) Caco2 cells were treated with IAld (10 μM) for 2 h, the AhR nuclear translocation was analyzed by immunofluorescence and quantified by Image J software, scale bar = 20 μm. (C) Molecular docking analysis of IAld binding on the ligand-binding domain of AhR protein. The analysis results indicated that IAld can form hydrogen bonds with amino acid residues (glutamine-118, arginine236). (D) Molecular docking results of IAld and AhR protein-binding pocket. (E, F) Caco2 cells were treated with IAld+TNF-α in the presence or absence of the AhR-specific inhibitor CH-223191 (10 μM) for 24 h, and the ZO-1 protein expression and cell membrane distribution were analyzed by immunofluorescence and quantified by Image J software, scale bar = 20 μm. (G) After the indicated treatment, the leakage of FD-4 induced by TNF-α-stimulation was measured. (H) Caco2 cells were treated with IAld in the presence or absence of the AhR-specific inhibitor CH-223191 (10 μM) for 24 h, and the p-AMPK and LC3 protein expression levels were measured by western blotting. (I, J) Caco2 cells were treated with IAld in the presence or absence of the CH-223191 for 24 h, and the interaction between AhR and p-AMPK protein was assayed by immunoprecipitation and quantified by Image J software. Data are presented as means±SEM (n = 3 or 4). * p<0.05, ** p<0.01, compared with the respective control. AhR, aryl hydrocarbon receptor; IAld, indole-3-carboxaldehyde; ZO-1, zona occludens-1; TNF-α, tumor necrosis factor-alpha; AMPK, AMP-activated protein kinase; SEM, standard error of the mean.

IAld is a known ligand of AhR [23], thus we examined the impact of IAld on AhR nuclear transport and activation. IF analysis revealed that IAld treatment (10 μM) significantly enhanced AhR nuclear transport in Caco2 cells (Figures 4A, 4B). Notably, even lower concentrations (1 μM) effectively promoted AhR nuclear activation (Supplements 2A, 2B). Molecular docking analysis further demonstrated that IAld binds to the AhR ligand-binding domain with a binding energy of −4.97 kcal/mol, confirming their strong interaction (Figures 4C, 4D).

To elucidate AhR’s role in IAld-mediated protection, we employed the AhR-specific inhibitor CH-223191. This inhibitor completely blocked IAld’s ability to maintain TJ integrity (Figures 4E, 4F) and its protective effect on intestinal barrier function in permeability assays (Figure 4G). Importantly, AhR inhibition also abolished IAld-induced AMPK signaling and downstream autophagy pathway activation (Figure 4H). The IP results revealed a physical interaction between AhR and p-AMPK protein (Figure 4I). Intriguingly, IAld treatment weakened this interaction, while CH-223191 enhanced it (Figures 4I, 4J). We propose that IAld promotes AhR nuclear translocation, thereby reducing its binding to p-AMPK. These above results suggest that IAld can exert intestinal barrier protection by activating the AhR/AMPK signaling.

### Indole-3-carboxaldehyde alleviates dextran sodium sulfate-induced colitis in an AMP-activated protein kinase-dependent manner

**Figure 5:**
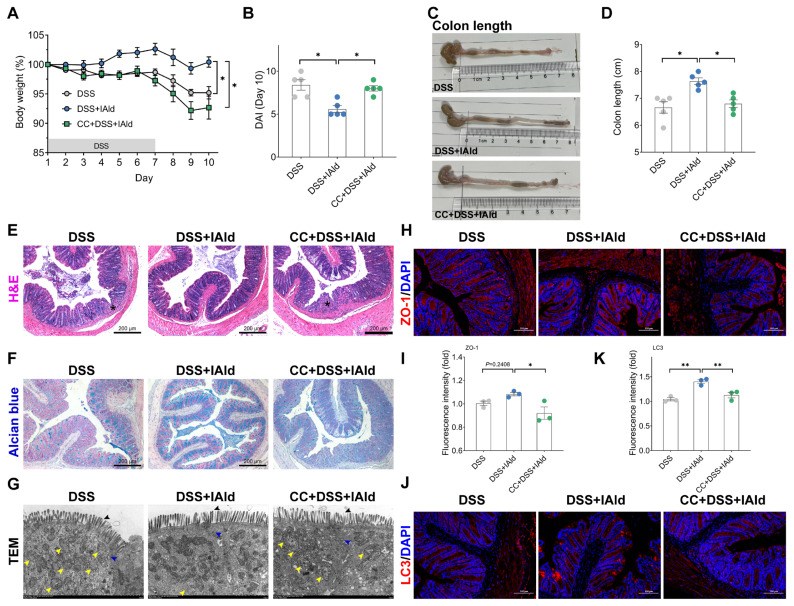
IAld alleviates DSS-induced colitis in an AMPK-dependent manner. (A) Body weight changes in colitis mice (n = 5). (B) DAI values at day 10 (n = 5). (C, D) Colon length and representative colon images (n = 5). (E, F) Representative images of colon H & E staining (asterisk: intestinal inflammatory infiltration and loss of intestinal crypts) and alcian blue staining (n = 3), scale bar = 200 μm. (G) Representative images of the microstructure of colonic epithelia by TEM (black arrows: brush border; yellow arrows: mitochondrial damage; blue arrows: tight junctions) (n = 3). (H, I) Representative images of immunofluorescence of ZO-1 distribution in the colon (n = 3), the ZO-1 protein expression was analyzed by immunofluorescence and quantified by Image J software, scale bar = 100 μm. (J, K) Representative images of immunofluorescence of LC3 puncta (marked with white arrows) in the colon (n = 3), the LC3 protein expression was analyzed by immunofluorescence and quantified by Image J software, scale bar = 100 μm. Data are presented as means±SEM. * p<0.05, ** p<0.01, compared with the respective control. DAI, disease activity index; DSS, dextran sodium sulfate; IAld, indole-3-carboxaldehyde; TEM, transmission electron microscopy; AMPK, AMP-activated protein kinase; ZO-1, zona occludens-1; SEM, standard error of the mean.

On the basis of clarifying that the AhR ligand IAld can exert barrier protection by activating AMPK signaling in intestinal epithelial cells, we further verified the role of IAld-induced AMPK activation *in vivo* (Supplement 3). The mouse colitis model was induced through 2.5% DSS administration, which was widely applicable to use as an intestinal barrier damage model [24]. As shown in Figures 5A, 5B, IAld (50 mg/kg body weight, this dose was selected based on our previously published literature [9]) exerted significant protective effect, as evidenced by promoted weight recovery and reduced DAI in DSS-induced colitis mice. Importantly, the colon length shortening, a critical pathological feature of colitis, was markedly alleviated by IAld treatment (Figures 5C, 5D). In addition, DSS-treated mice exhibited inflammatory cell infiltration and loss of goblet cells in the colon tissue, while the pathological morphology of the colon tissue in IAld-treated mice was relatively normal (Figures 5E, 5F). However, AMPK-selective inhibitor (Compound C, 1 mg/kg body weight, the concentration was selected based on published literature [25]) treatment almost completely blocked the protective effect of IAld on colitis, which can be determined by changes in mouse body weight, DAI, colon length, and histopathological analysis (Figures 5A–5F). The above results confirmed that the beneficial role of IAld was related to AMPK activation.

Finally, we verified the regulation of IAld on intestinal barrier and mitochondrial function in colitis mice. As shown in Figure 5G, we observed the ultrastructure of the intestinal epithelium through TEM and found that IAld can effectively alleviate DSS-induced mitochondrial damage (such as vacuolization, marked with yellow arrows). Besides, the colon epithelium from IAld-treated mice had a relatively fine structure of the TJs (marked with blue arrows) and brush border (marked with black arrows) (Figure 5G). However, Compound C treatment abolished these protective effects (Figure 5G). We further analyzed the effect of IAld on the TJ through IF analysis and found that IAld enhanced the expression levels of intestinal epithelial TJ protein ZO-1 (Figures 5H, 5I), while Compound C pre-treatment reduced the ZO-1 expression level (Figures 5H, 5I). In addition, IAld dramatically enhanced the LC3 protein expression levels in colon tissue, indicating an upregulation of autophagy level (Figures 5J, 5K). In summary, the above data confirmed that AhR ligand IAld can improve intestinal barrier and mitochondrial function in colitis mice, and the protective effect is related to AMPK-mediated autophagy induction.

## DISCUSSION

The integrity of intestinal barrier function is essential for maintaining intestinal homeostasis [26]. While previous studies have demonstrated that IAld can enhance TJ function in intestinal epithelial cells [7], the underlying regulatory mechanisms remain poorly understood. In this study, we systematically investigated the effects of IAld using both *in vivo* and *in vitro* intestinal inflammation models. Our findings reveal that IAld not only significantly improves mitochondrial function but also enhances intestinal barrier integrity in intestinal epithelial cells. Furthermore, we demonstrate that these beneficial effects are specifically mediated through the activation of the AhR/AMPK signaling. Taken together, these results collectively suggest that IAld represents a novel and promising therapeutic candidate for protecting intestinal barrier function and managing intestinal disorders in animals.

Previous studies have shown that Trp plays an important role in maintaining intestinal homeostasis in the body [27]. Interestingly, Trp-mediated barrier protection appears to be associated with the production of Trp metabolites by gut microbiota metabolism [28]. For example, Scott et al demonstrated that three types of gut microbiota-derived Trp metabolites can enhance intestinal epithelial barrier function and reduce the occurrence of intestinal inflammation in mice [7]. In addition, the intestinal colonization resistance induced by Trp is also mediated by its microbiota-derived indole derivatives [29]. In our study, we focused on a representative gut microbiota-derived Trp metabolite IAld, which serves as an important ligand for AhR and has been reported to have significant effects in alleviating colitis in mice [30]. We confirmed that IAld treatment enhances the expression level of TJ protein ZO-1 in intestinal epithelial cells Caco2 and mouse colon organoids (Figures 1A–1D), which corroborates existing studies [7]. TNF-α is an important pro-inflammatory cytokine known to impair intestinal epithelial barrier function and exacerbate intestinal inflammation [31]. We successfully constructed a model of intestinal epithelial barrier injury through TNF-α stimulation, and found that IAld treatment significantly alleviated the intestinal barrier damage caused by TNF-α *in vitro* (Figures 1E–1I). AMPK is a key molecule regulating biological energy metabolism and plays an important role in maintaining intestinal barrier function [11,32]. Notably, the regulatory role of AhR ligand IAld on AMPK signaling had not been clearly elucidated. Strikingly, we observed that IAld significantly enhances AMPK phosphorylation in intestinal epithelial cells, both under basal conditions and in the presence of TNF-α stimulation (Figures 2A–2D). To further validate the functional relevance of this finding, we employed the AMPK-specific inhibitor Compound C, which completely abolished the barrier-protective effects of IAld (Figures 2E–2I). These results collectively establish that AMPK serves as a critical pathway through which IAld exerts its protective effects against intestinal inflammatory diseases.

Autophagy is an evolutionarily conserved metabolic process in which cells form autophagosomes to encapsulate damaged organelles (such as damaged mitochondria) or abnormal proteins, which are then transported to lysosomes for degradation and recycling [33]. Studies have shown that autophagy is crucial for maintaining the gut barrier function [20,34], and it can inhibit inflammation-induced apoptosis of intestinal epithelial cells and maintain the integrity of the intestinal barrier [35]. In addition, it can directly enhance TJ barrier function by targeting the claudin-2 protein [36]. AMPK is the upstream signal that induces autophagy and plays a crucial regulatory role in maintaining mitochondrial homeostasis. Impaired autophagy levels or mitochondrial dysfunction can lead to the occurrence of intestinal inflammation [37,38]. In the present study, we found that IAld can enhance the level of autophagy and maintain mitochondrial function in intestinal epithelial cells by activating AMPK signaling, while AMPK inhibition can block the effect of IAld-mediated TJ barrier and mitochondrial function enhancement both *in vivo* and *in vitro* (Figures 3, 5). These findings further confirm that IAld enhances intestinal barrier function through AMPK activation.

AhR is a key transcription factor for maintaining barrier function and regulating intestinal homeostasis [39]. A recent study demonstrated that gut microbiota-derived 3-phenylpropionic acid can activate AhR, thereby promoting the intestinal epithelial barrier function [6]. Although the literature suggests that AhR can utilize cellular zinc signals to maintain the gut barrier [12], the impact of AhR on intracellular signaling pathways (such as AMPK) related to intestinal barrier function remains unclear. In the regulation of skin barrier function, human β-Defensin-3 can activate the autophagy pathway through the AhR signal and enhance the skin TJ barrier, ultimately alleviating atopic dermatitis-like inflammation [20], indicating a correlation between AhR and cellular autophagy function. In this study, we demonstrated that the AhR-specific inhibitor CH-223191 effectively blocks IAld-induced AMPK activation, autophagy enhancement, and TJ barrier promotion in intestinal epithelial cells, suggesting that IAld activates AMPK through AhR nuclear translocation (Figures 4A–4H). Notably, we identified a physical interaction between AhR and p-AMPK proteins, and found that IAld attenuates this interaction (Figures 4I, 4J). These findings provide, at least in part, mechanistic insights into how IAld activates AMPK, highlighting the role of AhR in this regulatory process.

## CONCLUSION

**Figure 6:**
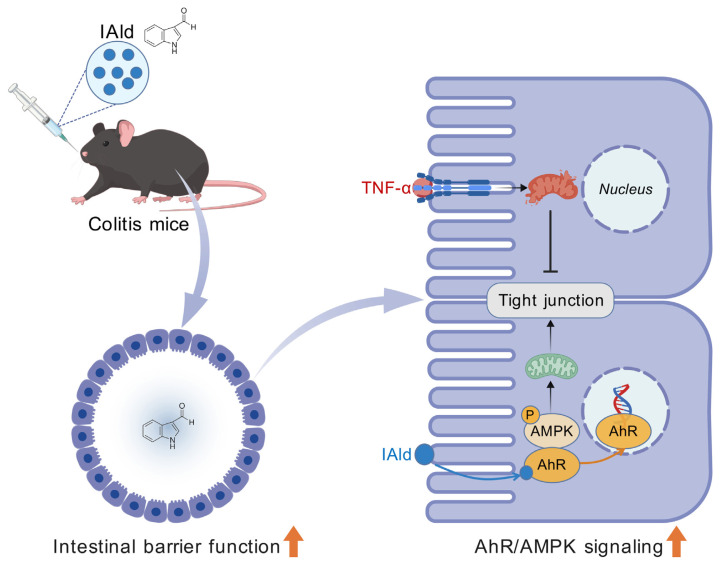
Schematic illustration of the proposed mechanism by which IAld ameliorates intestinal barrier dysfunction. IAld activates the AhR/AMPK signaling pathway, which in turn maintains mitochondrial homeostasis in intestinal epithelial cells and alleviates intestinal barrier dysfunction in colitis mice. This schematic diagram was created with BioGDP.com. IAld, indole-3-carboxaldehyde; TNF-α, tumor necrosis factor-alpha; AMPK, AMP-activated protein kinase; AhR, aryl hydrocarbon receptor.

In conclusion, our study reveals that the gut microbiota-derived Trp metabolite IAld significantly ameliorates intestinal barrier dysfunction in both *in vitro* and *in vivo* models. Mechanistically, IAld activates the AhR/AMPK signaling pathway, preserves mitochondrial functional homeostasis in intestinal epithelial cells, and thereby enhances intestinal barrier integrity (Figure 6). These findings establish IAld as a crucial endogenous protective factor for intestinal barrier function, highlighting its therapeutic potential for intestinal inflammatory diseases in animals.

## SUPPLEMENTARY MATERIAL

**Supplement 1.** IAld does not alter AMPK protein abundance in Caco2 cells.

**Supplement 2.** IAld activates AhR signaling in Caco2 cells.

**Supplement 3.** Schematic of animal experiments (n = 5).
