# Supplementary material for: Gut microbiota-derived tryptophan metabolite indole-3-carboxaldehyde enhances intestinal barrier function via aryl hydrocarbon receptor/AMP-activated protein kinase signaling activation
Source: Anim Biosci. 2025 Jul 11;39(1):250225. doi: 10.5713/ab.25.0225 (PMC12754468; doi:10.5713/ab.25.0225)
Supplement: Supplementary file 1 [file ab-25-0225-Supplementary-1.pdf]

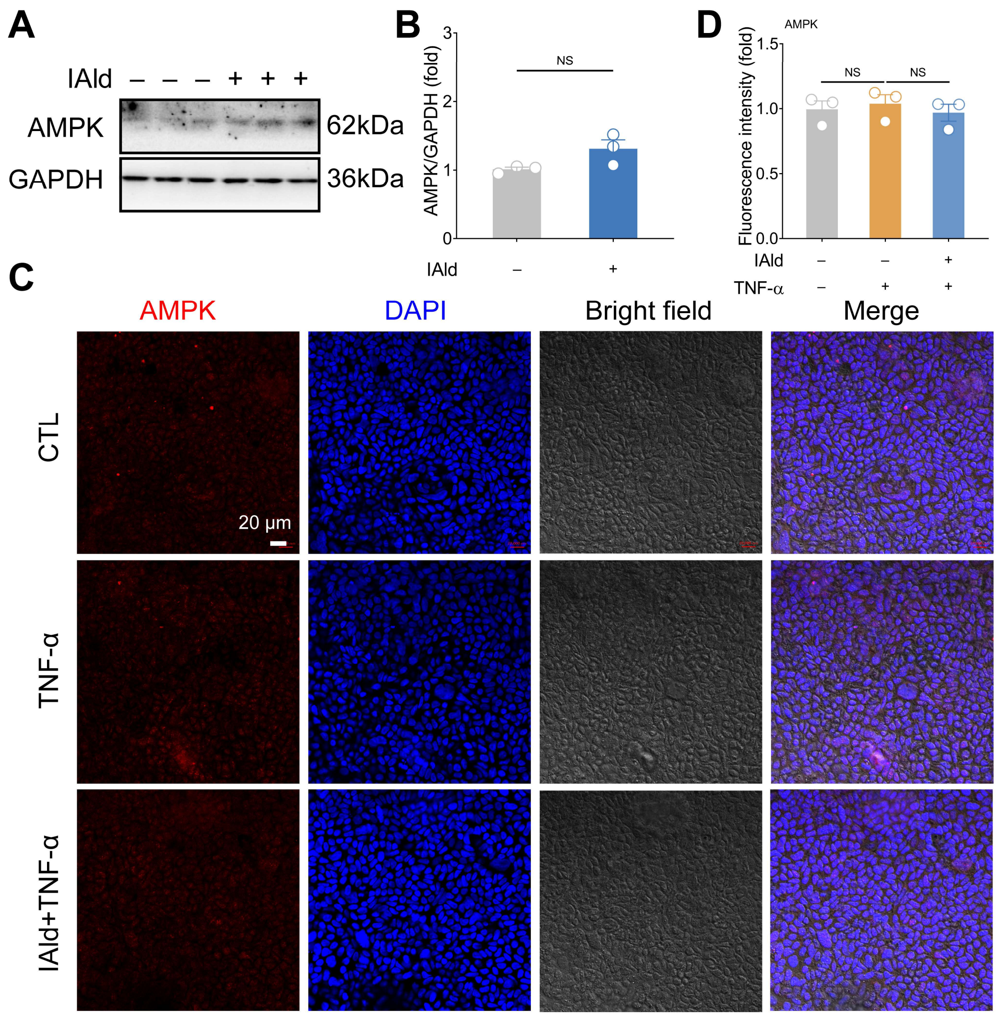

Supplement 1. IAld does not alter AMPK protein abundance in Caco2 cells.

Related to Figure 2

(A, B) Caco2 cells were treated with IAld (0, 10 μM) for 24 h, the AMPK protein expression levels were measured by western blotting and quantified by Image J software. (C, D) Caco2 cells were treated with IAld and stimulated with TNF-α (10 ng/mL) for 24 h. The AMPK protein expression was analyzed by immunofluorescence and quantified by Image J software, scale bar = 20 μm. Data are presented as means ± SEM (n=3). NS: no significance between the indicated groups.
