# Supplementary material for: Gut microbiota-derived tryptophan metabolite indole-3-carboxaldehyde enhances intestinal barrier function via aryl hydrocarbon receptor/AMP-activated protein kinase signaling activation
Source: Anim Biosci. 2025 Jul 11;39(1):250225. doi: 10.5713/ab.25.0225 (PMC12754468; doi:10.5713/ab.25.0225)
Supplement: Supplementary file 2 [file ab-25-0225-Supplementary-2.pdf]

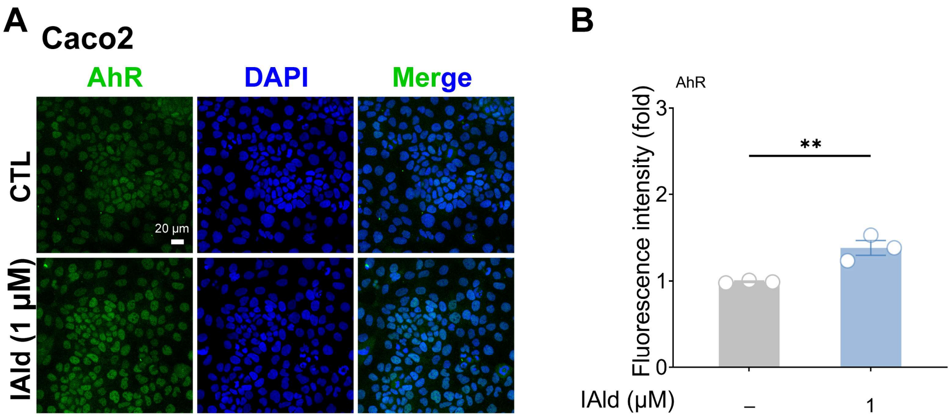

Supplement 2. IAld activates AhR signaling in Caco2 cells.

Related to Figure 4

(A, B) Caco2 cells were treated with IAld (1 μM) for 2 h, the AhR nuclear translocation was analyzed by immunofluorescence and quantified by Image J software, scale bar = 20 μm. Data are presented as means ± SEM (n=3). ***P* < 0.01, compared with the respective control.
