# Supplementary figures and images for: Gut microbiota-derived tryptophan metabolite indole-3-carboxaldehyde enhances intestinal barrier function via aryl hydrocarbon receptor/AMP-activated protein kinase signaling activation
Source: Anim Biosci. 2025 Jul 11;39(1):250225. doi: 10.5713/ab.25.0225 (PMC12754468; doi:10.5713/ab.25.0225)

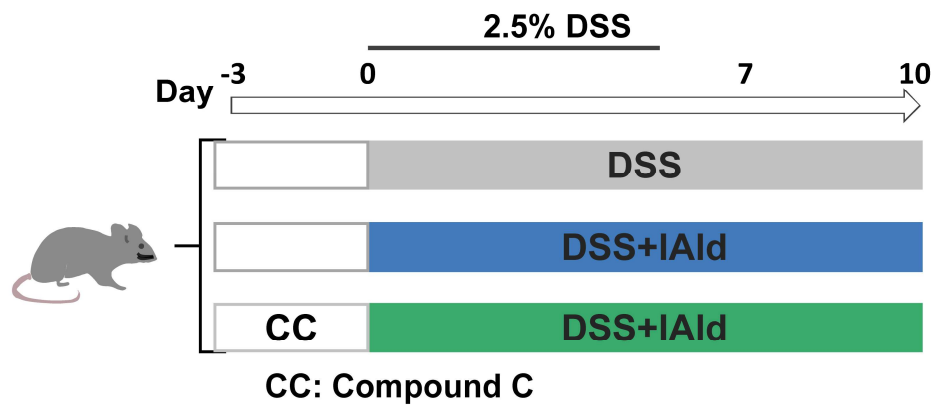

Supplement 3. Schematic of animal experiments (n = 5).

Related to [Figure 5](#)

Supplement: Supplementary file 3 [file ab-25-0225-Supplementary-3.pdf]
